# Niagara^@^ catheter equipped with a novel helical flow inducer to improve hemodynamic performance

**DOI:** 10.3389/fbioe.2025.1545996

**Published:** 2025-03-13

**Authors:** Yang Yang, Hao Yan, Huang Xianli, Ran Maoxia, Liu Chen, Liu Zhuang, Chen Yu, Zhang Ling

**Affiliations:** ^1^ College of Architecture and Environment, Sichuan University, Chengdu, Sichuan, China; ^2^ Department of Nephrology, Zigong First People’s Hospital, Zigong, Sichuan, China; ^3^ Department of Nephrology, Dazhou Central Hospital, Dazhou, China; ^4^ Department of Nephrology, Kidney Research Institute, West China Hospital of Sichuan University, Chengdu, Sichuan, China; ^5^ College of Chemical Engineering, Sichuan University, Chengdu, Sichuan, China

**Keywords:** catheter, hemodynamic, helical flow, local normalized helicity, residence time

## Abstract

Hemodialysis is an important means to sustain life in patients with end-stage renal disease In China, more than 100,000 hemodialysis patients need to have a catheter fitted at least once (temporary or long term) for dialysis. Despite the widespread use and low cost of HD catheters, they remain prone to critical issues such as high thrombosis rates, infections, and dysfunction. This study addresses the persistent challenge of thrombosis formation in dialysis catheters by investigating the incorporation of helical flow inducers, a strategy inspired by the naturally occurring helical blood flow in arterial systems. In this research, helical flow inducers with varying pitch and diameter were integrated into the widely used Niagara^@^ catheter. Computational fluid dynamics simulations were conducted to evaluate the impact on key parameters such as local normalized helicity (LNH), residence time (RT), shear stress, and flow velocity. The results demonstrated that 1) small-diameter inducers produce helical flow. Among inducers with identical diameter, those with a smaller thread pitch are more likely to induce increased LNH; 2) a small thread pitch helical flow inducer reduced the percentage of blood volume, with RT exceeding 0.015 s from 40.8% in the control to 12.7%, suggesting a substantial reduction in thrombosis risk; 3) the study also found that the introduction of small thread pitch helical flow inducers led to increased shear stress, with Model A showing an average shear stress of 49.2 Pa, compared to 32.0 Pa in the control. This highlights the need for careful optimization to balance the benefits of reduced thrombosis risk with the potential for shear-induced hemolysis. In conclusion, the integration of helical flow inducers into dialysis catheters offers a promising strategy for improving intraluminal flow dynamics and reducing the risk of thrombosis.

## 1 Introduction

Hemodialysis (HD) is a kidney replacement method for the treatment of end-stage renal disease (ESRD) (Xu et al., 2020). For dialysis treatment, building an ideal vascular pathway is the primary preparation before treatment, and it is also a necessary condition to successfully achieve treatment effects (Zhang et al., 2021). In China, more than 100,000 hemodialysis patients need to have a double-lumen catheter fitted at least once (temporary or long-term) for dialysis treatment (Yang et al., 2021). HD with double-lumen catheters may cause a major complication: thrombus or fibrin sheath formation around the catheter (Langston and Eatroff, 2018; Peng et al., 2017). However, the exact mechanism underlying catheter-associated thrombosis is still unclear. In recent years, some contributing factors have been suggested: stagnant blood zones, platelet adhesion in the catheter lumen, and regions of elevated shear stress that induce platelet activation may promote the formation of a blood clot (Lihua et al., 2022; Pinelli et al., 2021; Thapa et al., 2016). Significant levels of stagnating blood zones are mostly present in the blood-withdrawing (arterial) lumen of the catheter, more specifically in the distal tip region. Further study of the local hemodynamics of different catheter designs might provide insights into how catheters can be optimized to reduce the levels of stagnant blood zones, thus possibly leading to the use of fewer thrombogenic catheters.

Helical blood flow has been observed in several arteries and may be a normal physiological flow phenomenon of the arterial system (Morbiducci et al., 2011). Preliminary studies demonstrated that the widely existing helical flow may have several positive physiological roles, such as stabilizing blood flow transport, reducing blood flow disturbance, enhancing mass transport, and suppressing platelet and monocyte adhesion (Kwak et al., 2024; Zhan et al., 2010; Gallo et al., 2012). In recent years, introduction of helical flow has been shown to improve the hemodynamic performance of vascular devices such as arterial grafts, stents, and arteriovenous shunts (Liu et al., 2015) and to overcome flow-induced thrombus formation and intimal hyperplasia. For instance, Caro et al. (2005) designed small-amplitude helical grafts (Swirl Graft) and self-expanding helical stents to generate physiological-type helical flows for reducing thrombus formation and intimal hyperplasia in artery bypass grafts and stented arteries. In addition, Stonebridge et al. (2012) proposed a type of spiral laminar flow graft with spiral folds on the endo-luminal surfaces known to reduce the disturbed flow at the distal anastomosis in arterial bypass grafts and arteriovenous shunts. Zheng et al. (2014) numerically simulated the hemodynamic characteristics of several arteriovenous grafts with various helical shapes, and their simulation results indicated that helical grafts suppress the disrupted shear stress distribution in the venous segment compared to the conventional straight graft. Chen et al. (2018) also demonstrated that use of a novel helical flow vena cava filter design, compared with the traditional filter, could not only induce helical flow but could also partly reduce the RT.

In this study, inspired by the use of helical flow in artificial blood vessels and venous filters to improve their hemodynamic performance, we proposed that helical flow inducers be applied to dialysis catheters to reduce their risk of thrombosis; moreover, the new catheter designs exhibit lower residence time (RT) and higher local normalized helicity (LNH) values than commercial Niagara catheters without inducing excessive shear stress. Specifically, our analysis considered the pitch and diameter of helical flow inducers, RT and LNH, and the damage caused to blood cells by shear stress, which are crucial parameters that determine the probability of thrombus formation within catheters. The finding provides new insights for future structural optimization design of catheters.

## 2 Methods

### 2.1 Geometry of hemodialysis catheters with helical flow inducers

**FIGURE 1:**
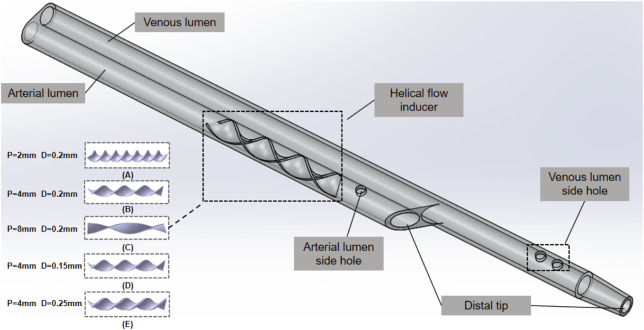
Niagara^@^ catheter.

The Niagara catheter, as a commercially available hemodialysis catheter, is widely used. Some studies have shown that the Niagara catheter posed the lowest resistance to flow and higher median circuit life (11 h > 7 h) than the Medco catheter (Fealy et al., 2018). The Niagara catheter, as shown in Figure 1, exhibits a step tip design, and the inner diameter of the arteries and venous lumen is 2 mm. The two circular side-holes with a vertical distribution and distal tips with nozzle shapes are located on the venous lumen. One circular side-hole for the inflow is located on the arterial lumen. All these circular side holes’ diameters are 1 mm. The helical flow inducer is located on the arterial lumen.

**FIGURE 2:**
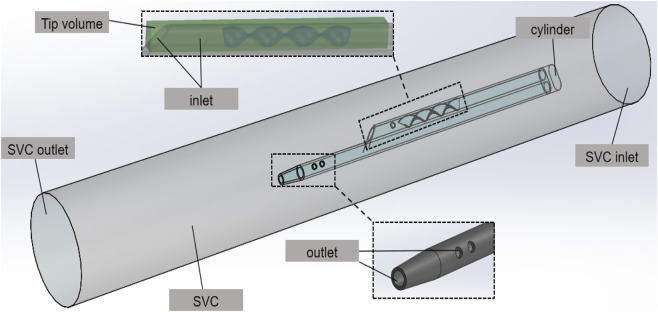
3D model of the SVC and the catheter.

The catheter with helical flow inducer model consists of a helical flow inducer and a Niagara^@^ catheter, which was created using the computer-aided design software SolidWorks software (Dassault System SolidWorks, Concord, MA). The following models are considered: models A–C, in which the inducer has an invariable diameter of 0.2 mm, and the thread pitch values of the helical flow inducers were 2, 4, and 8 mm, respectively; models D–E, in which the inducers have the same thread pitch of 4 mm, and the diameters of the helical flow inducers were 0.15 and 0.25 mm, respectively. The length of the inducer was 16 mm, and the entire arterial lumen length was 40 mm. In the model domain, the 3D model of the superior vena cava (SVC) and the catheter is shown in Figure 2. The SVC is cylindrical with a diameter of 20 mm. In order to eliminate the impact of unrelated variables on the performance, the lengths of the SVC and each catheter are set to 150 mm and 70 mm, respectively and the distance between the distal tips of the catheter and the SVC is fixed to 55 mm so that the blood fully normalizes inflows to the catheter in order to develop and no outlet effects to occur.

In the SVC fluid domain, a cylindrical cavity of length 3 mm and the same diameter as the outer diameter of the catheter is dug out at the front end of the root catheter, thus imposing a boundary condition. In order to better compare inflow parameters, a cuboid “tip volume” is defined at the tip of the venous lumen for each catheter. Its width and height are equal to the outer diameter and radius of the catheter, respectively, and it includes the entire inflow lumen. In order to focus on the flow characteristics of the different catheters, the tip volume is extended from the most distal point of the catheter up to 30 mm, where the flow velocity has become stable and fully developed, with no further flow interference.

### 2.2 Mesh of hemodialysis catheters

**FIGURE 3:**
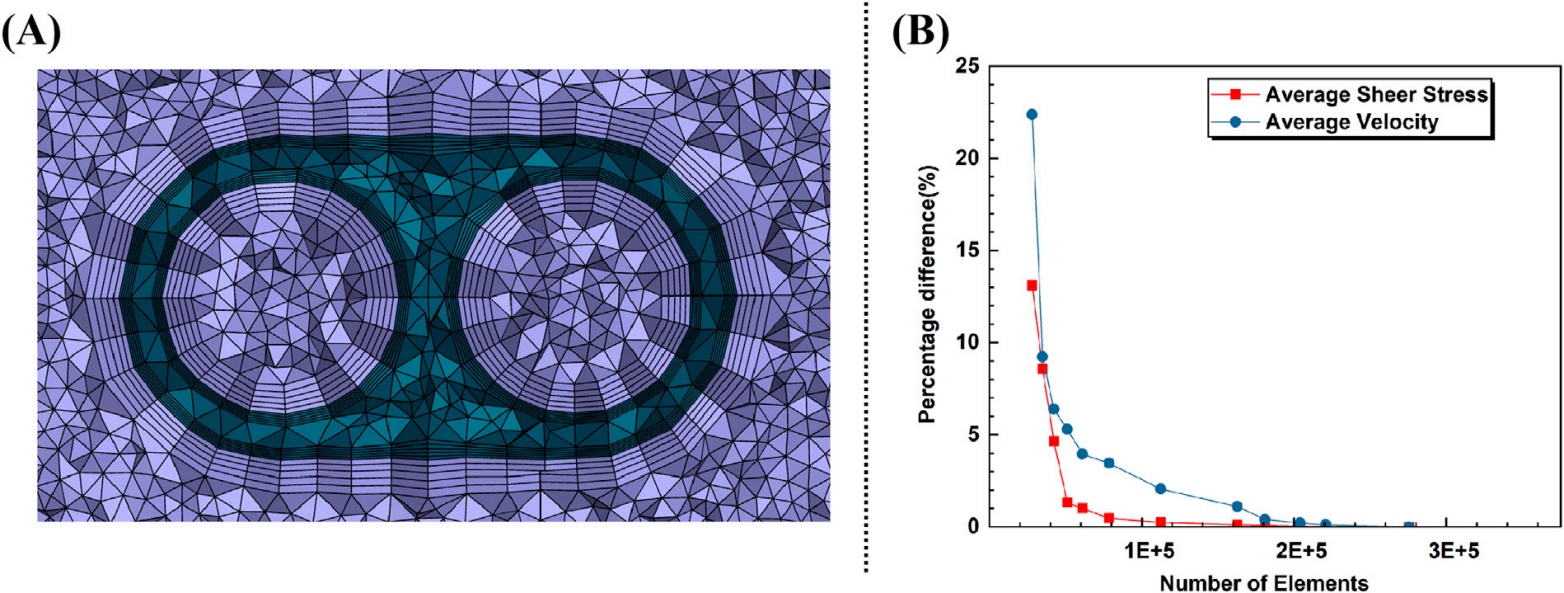
**(A)** Cross-section of the grid. **(B)** Percentage difference with increasing elements for average velocity and shear rate in tip.

We performed simulations on the model-C design, increasing the mesh density incrementally at twelve points. The mesh used in the study was chosen once the percentage difference for average velocity and average shear stress in the tip was below 0.5%. This resulted in a 210,543-element predominantly tetrahedral mesh, with five prismatic layers surrounding the inner/outer lumens of the catheter body. The average skewness and orthogonal quality were 0.35 and 0.64, respectively. According to common computational fluid dynamics (CFD) mesh quality criteria, a skewness below 0.5 and an orthogonal quality above 0.3 are considered acceptable. Therefore, the mesh meets these standards, ensuring reliable simulation results. In addition, a cross-section of the grid is shown in Figure 3A, and the percentage differences for the varying mesh densities are shown in Figure 3B.

### 2.3 Governing equations and boundary conditions

The simulations for flow motion were based on the three-dimensional incompressible Navier–Stokes equations (Meirson et al., 2015) as shown in Equations 1, 2:

$$\rho \left(\left(\partial_{\nu }/\partial_{t}\right)+\left(\nu \cdot \nabla \right)\nu \right)=-\nabla p+\nabla \cdot \tau ,$$ (1)

∇⋅ν=0, (2)

where ν and ρ , respectively, represent the fluid velocity vector and the pressure. The density of blood was taken as ρ = 1,050 kg/m3. The fluid is blood, which is identified as an incompressible, uniform, non-Newtonian fluid (Carty et al., 2016), the viscosity of which varies with the shear rate. In previous studies, there were established incompressible Newtonian fluid models as the working fluid, whereas this study considered the change in the shear rate occurring during inflow and used an asymptotic shear thinning Carreau model (Lee and Steinman, 2007), which was defined using the shear rate in Equation 3, the stress tensor in Equation 4, and defined by Equation 5.

$$\dot{\gamma }=\sqrt{2*D_{ij}\cdot D_{ij},}$$ (3)

where $\dot{\gamma }$ is the shear rate and D is the strain rate tensor with i, j = 1,2,3 as the inner projects.

$$\tau =\mu \left(\dot{\gamma }\right)\cdot \left(\nabla \nu +{\left(\nabla \nu \right)}^{T}\right),$$ (4)

where μ is the viscosity of blood and τ is the stress tensor, with ∇ν being the gradient of the fluid velocity.

$$\mu =\mu_{\infty }+\left(\mu_{\infty }+\mu_{0}\right){\left[1+{\left(\lambda \dot{\gamma }\right)}^{2}\right]}^{\frac{n-1}{2}},$$ (5)

where $\mu_{\infty }$ = 0.00345 Pa.s, n = 0.25, $\mu_{0}$ = 0.025 Pa.s, and λ = 25 s.

Residence time at any location indicates the time that has elapsed since the fluid in that location has entered the lumen. As the flow inside the catheter lumen is proven to be three-dimensional, shear stress and blood residence time were evaluated in a constant tip volume of the arterial lumen of each catheter model. A tip volume of 0.075 mL was considered, corresponding to the distal 23.8 mm end of the arterial lumen in the Niagara catheter. Volume-averaged shear stress and residence time in this tip zone are calculated for all catheters.

LNH is a dimensionless parameter that describes the alignment between velocity and vorticity vectors while reflecting the intensity of their interaction, providing a quantitative measure of the presence and strength of helical flow structures. (Morbiducci et al., 2009) as defined in Equation 6:

$$\begin{array}{cc} LNH=\frac{\vec{u}\cdot \vec{\omega }}{\left|\vec{u}\right|\left|\vec{\omega }\right|} & -1\leq LNH\leq 1, \end{array}$$ (6)

where v and ω represent the fluid velocity vector and vorticity, respectively. The LNH is the local value of the cosine of the angle between the velocity and vorticity vectors, describing the alignment (how closely they point in the same or opposite direction) and intensity (the strength of their interaction based on their magnitudes). Theoretically, the smaller the absolute value of LNH, the greater the tendency toward purely axial flow; conversely, a larger absolute value indicates a tendency toward swirling flow. It can be inferred that a large and continuous region with high LNH indicates the helical flow. In contrast to purely axial flow, Zhan et al. (2010) investigated the impact of swirling flow on platelet adhesion along the glass tube wall. The experimental study’s findings showed that the swirling flow produced in the test tube significantly decreased platelet adhesion to the test tube’s surface.

#### 2.3.1 Boundary conditions

The SVC inlet is set to a 0.3 m/s velocity-inlet, and the outlet is a pressure-outlet with a gauge pressure of 0. The surfaces of the catheter and SVC are set as the wall without slip condition, and the inlet and outlet of the catheter are set as the mass-flow-inlet and mass-flow-outlet of 400 mL/min, respectively (Yang et al., 2022).

#### 2.3.2 Solution settings

The CFD software (ANSYS FLUENT 2022 R1, ANSYS, Inc., Canonsburg, PA, USA) COUPLED solver was used for numerical simulation of the fluid. The flow in the catheter was similar to that observed in other studies on catheters (Clark et al., 2015; Owen et al., 2020; Cho et al., 2021), assuming laminar flow. The mass continuity residual magnitude is less than 10^−6^, and the combined flow rate of the tip and the side-hole equal to 400 ± 1 mL/min were used to assess convergence as criteria.

## 3 Results

### 3.1 Helicity

**FIGURE 4:**
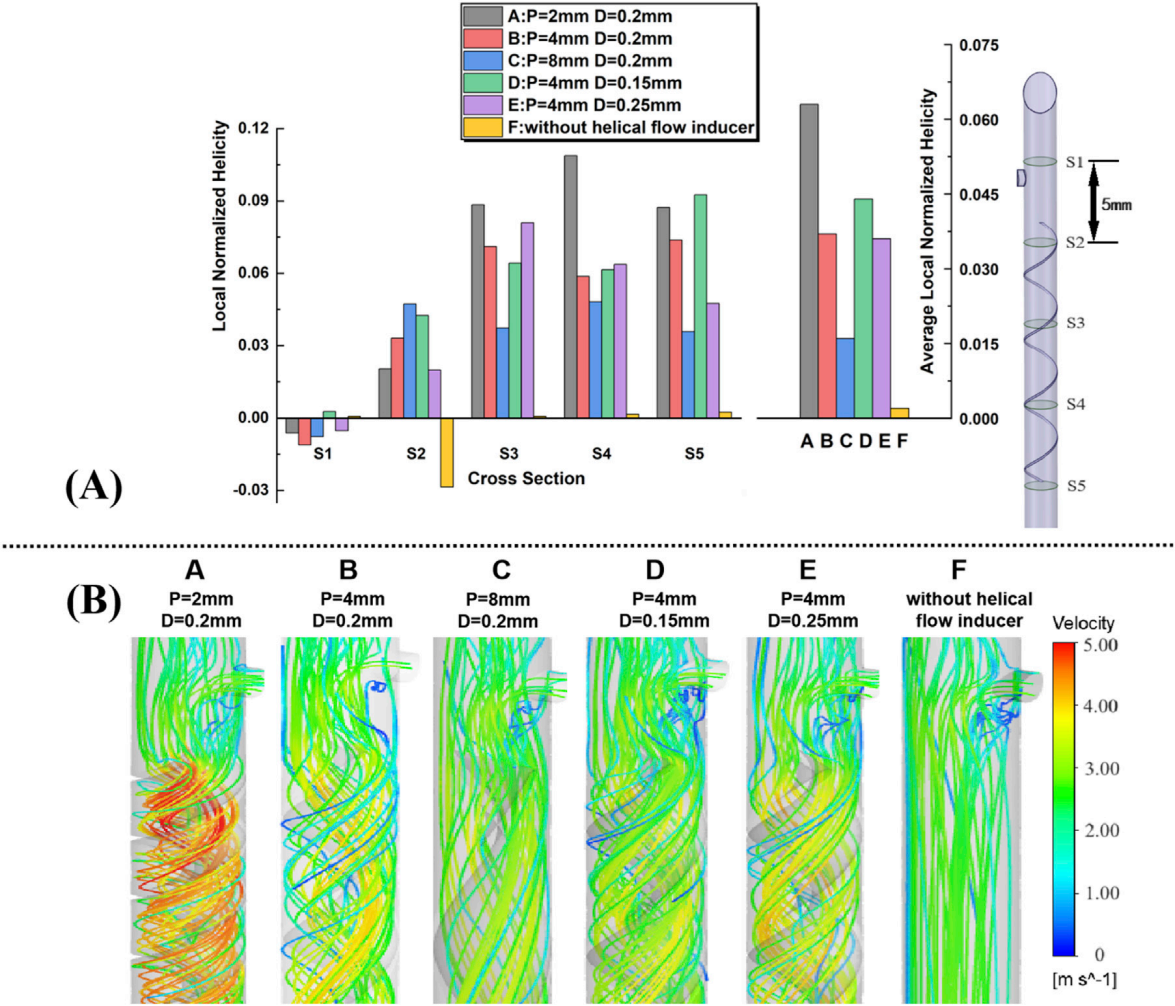
**(A)** Five representative slices in the catheter model, with adjacent slices spaced 5 mm apart; plots of area-weighted averages from steady flow computations of local normalized helicity (LNH) for all the five slices for each case. **(B)** Velocity streamlines near the inducers are obtained from the steady flow computations.

A comparative analysis of local normalized helicity (LNH) across various catheter designs incorporating different helical flow inducers is shown in Figure 4A. Five representative cross-sections (S1–S5) along the catheter models were selected to plot area-weighted averages of LNH, providing insights into the influence of helical flow inducers on internal flow dynamics. The results indicate that all helical flow inducers (Cases A to E) successfully induce non-zero helicity within the catheter, in sharp contrast to the control case (Case F), where helicity values are nearly 0, with some sides, particularly Side 2, even exhibiting negative helicity. The negative LNH observed in the control case, especially at S2, indicates that in the absence of helical flow inducers, localized misalignment between the velocity and vorticity vectors occurs in certain regions. This misalignment, where the vectors point in opposite directions, arises from flow disturbances in these areas. Over time, such disturbances could increase the risk of adverse hemodynamic effects, including flow instability and potential zones of stagnation, which are known contributors to thrombosis. Notably, a comparison between Cases D and E, which share identical pitch but have different diameters, reveals that a smaller diameter results in a marginally higher LNH. Additionally, across Cases A, B, and C, which vary in pitch, it is evident that increasing the pitch of the helical flow inducer tends to reduce the overall helicity, suggesting that more tightly wound inducers enhance the helicity. The relative helicity distribution graph highlights that all helical inducers significantly increase helicity compared to the control, with Case A (featuring a 2 mm pitch and 0.2 mm diameter) achieving the highest helicity values across multiple cross-sections. Furthermore, the average relative helicity across models reaffirms that configurations with smaller pitches or larger diameters produce varying degrees of helicity, with Case A again demonstrating most enhancement in helicity. These findings underscore the potential for optimizing helical flow inducer design to improve catheter performance by promoting uniform helical blood flow, thereby mitigating thrombosis risk.

### 3.2 Streamlines

Velocity streamlines from steady-state simulations near the helical flow inducer in the five models are shown in Figure 4B, with color coding for the velocity magnitude (speed). For comparison, the model without a helical flow inducer is also depicted. In model-F, except for the area near the side hole, throughout the entire arterial lumen, there is no significant change in blood flow velocity. However, the increase in the flow velocity in the lumen center is more pronounced after the introduction of the helical flow inducer. In model A, helical flow is more evident, while in model D, the streamlines are smoother. In addition, the flow velocity is, in general, higher at the inducer’s center in models A and B.

### 3.3 Residence time

Blood cell RT was calculated in the same tip volume by solving a specific form of the continuity equation as in a previous study (Mareels et al., 2007). This RT value indicates the time for which blood remained in the arterial inlet volume before being drained to the arterial outlet, i.e., the ‘‘washout time’’ of blood in the arterial inlet and a proxy of the risk of catheter tip thrombosis. Concerning the residence time, the average value of parameter t at the end of the tip volume is 0.015 s for the Niagara catheter, given the tip volume and the catheter blood flow rate. However, due to local stagnation of blood in the tip zone, the time since the blood has entered the arterial lumen can locally exceed 0.015 s (which occurs in regions close to the catheter wall and in areas where blood flow is disturbed). Consequently, the percentage of tip volume with RT over 0.015 s was evaluated for each catheter design.

**FIGURE 5:**
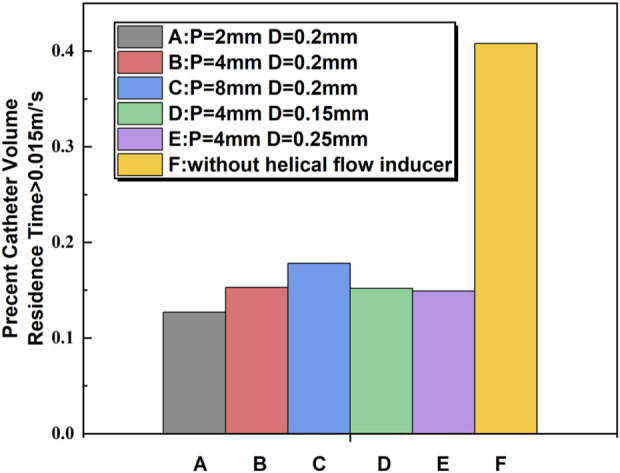
Comparison of blood RT > 0.015 s between the catheters calculated from computational flow dynamics as a percentage of flow through the catheter.

The percent volume of blood flowing through the catheter with an RT exceeding 0.015 s was calculated. It is shown that the Niagara^@^ catheter design produced a percent volume of RT > 0.015 s of 40.8%, compared to 15.3% for the Niagara^@^ catheter with a 0.2-mm-diameter and 4-mm-pitch helical flow inducer (model-B). This is compared to a percent volume of RT > 0.015 s of 17.8% for the inducer of 8 mm pitch (model-C). The lowest RT was present with the inducer with 2 mm pitch (model-A), which was associated with a 12.7% percent volume of RT > 0.015 s. As shown in Figure 5, the change in the diameter of the helical flow inducer has little effect on the proportion of the area with a velocity greater than 0.015 m/s.

### 3.4 Shear stress and velocity

**FIGURE 6:**
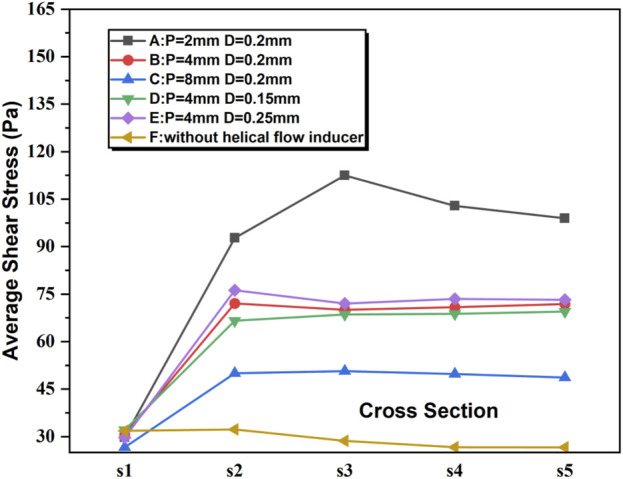
Shear stress for the five representative slices of the catheter in the steady flow computations.

The shear rate profiles for the five examples of catheter cross-sections utilizing the various models are displayed in Figure 6. In all the models, the shear stress of the blood flow adjacent to the inducers is much higher relative to other regions within the catheter. Near the inducer, models A–E evidently induce progressively higher shear stress at spatial locations ranging from S2 to S5, with the maximum shear rate value elicited by model A in S3. In comparison, the shear rates for models C and D are lower. In S1, there is no evident difference between the different models A–F.

It is believed that a velocity of less than 0.1 m/s would cause blood retention and lead to thrombosis (Wong et al., 2014). The maximum flow rate, the average shear stress, and the proportion of the area of velocity under 0.1 m/s were extracted from the tip volume of these six catheters to quantitatively assess the hemodynamic performance of each catheter.

It is shown that the percentage of velocity regions (0.1 m/s) was 1.22%, and the maximum flow velocity and average shear stress at the tip volume of a normal Niagara catheter were 3.583 m/s and 34.395 pa, respectively. As shown in Table 1, both the average flow velocity and the maximum flow velocity within the tip volume of each catheter increased to varying degrees, for example, model B, where the diameter and thread pitch of the inducer were 0.2 mm and 4 mm, respectively, with the average flow velocity increasing from 1.824 m/s to 2.433 m/s, and the maximum flow velocity also increased to 4.42 m/s, and the regions where the velocity at the tip volume was 0.1 m/s showed little change. Corresponding to the velocity levels, the average shear stress within the tip volume also increased accordingly to 36.024 Pa. When the diameter of the helical flow inducer was kept the same at 0.2 mm and the pitch was changed from 4 mm to 2 mm and 8 mm, the flow velocity and shear stress level in the tip volume changed. As the pitch of the helical flow inducer increases, both the maximum flow velocity (5.624 m/s < 4.42 m/s < 3.888 m/s) and the average flow velocity (2.909 m/s < 2.433 m/s < 2.291 m/s) within the tip volume of the catheter’s arterial lumen decrease, which indicates that a helical flow inducer with a smaller pitch has a positive effect on increase in the flow velocity levels within the catheter. With an increase in the pitch of the helical flow inducer, the shear stress at the tip volume in the arterial lumen changed significantly, with average shear stress decreasing from 49.203 pa to 36.024 pa and 32.298 pa. It was presumed that increasing the pitch of the inducer would reduce the shear stress in the tip volume of the arterial lumen.

**TABLE 1:** Maximum flow velocity and average shear stress at the tip volume of six catheters.

	Max velocity (m/s)	Average velocity (m/s)	Velocity <0.1 m/s	Average shear stress (Pa)
A	5.624	2.909	0.13%	49.203
B	4.418	2.433	0.43%	36.024
C	3.888	2.291	0.89%	32.298
D	4.259	2.378	0.61%	34.762
E	4.561	2.494	0.51%	37.664
F	3.583	1.824	1.22%	32.001

When the pitch of the helical flow inducer was kept constant at 4 mm and the diameter of the inducer was changed from 0.15 mm to 0.2 mm and 0.25 mm, it is observed that as the diameter decreases, both the average flow velocity (2.378 m/s < 2.433 m/s < 2.494 m/s) and the maximum flow velocity (4.259 m/s < 4.418 m/s < 4.561 m/s) within the tip-volume of the catheter’s arterial lumen correspondingly decrease, along with a reduction in the average shear stress. It is speculated that a helical flow inducer with a larger diameter has a positive effect on enhancing the flow velocity within the catheter.

## 4 Discussion

Recently, several studies have demonstrated the beneficial effects of helical flows in arterial bypass surgery, arterial stenting, and in the venous system. Within a computational flow model of arterial stenosis, spiral flow was associated with a 700% decrease in near wall turbulent energy compared to nonspiral flow (Houston et al., 2004). In addition, variation in shear stress has been identified as a risk factor for thrombogenicity through platelet activation. Clearance of blood from the catheter tip is also considered a critical component in catheter design; catheters with higher levels of blood RTs (exceeding 0.015 s) (Mareels et al., 2007) and blood stagnation regions (velocity<0.1 m/s) (Wong et al., 2014) in computational flow models have been considered susceptible to thrombus formation through platelet activation and delayed washout of blood near the wall of the catheter lumen.

Due to the versatile physiological functions of helical flow, intentionally inducing a helical flow in a catheter arterial lumen is likely to assist in overcoming the challenges of thrombus formation. In the present study, we proposed a novel catheter design which helical flow is induced in the arterial lumen to improve the local hemodynamics of the catheter. To evaluate this design, numerical simulations were carried out to compare catheters with helical flow inducers with various thread pitches and various diameters under steady flow conditions.

Comparison of the numerical simulation results from these cases demonstrated that the helical flow inducer with a small thread pitch induced higher helicity and decreased the high levels of blood RTs. Furthermore, our numerical simulation found that compared with the catheter without the inducer (Case F), the new design, particularly the catheter with a smaller-thread-pitch inducer (Case A), not only induces helical flow but also partly increases maximum and average velocity in the catheter vicinity. However, given the blood flow in the catheter, changes in the fluid shear stress also have an influence on the development of thrombosis. Wang proposed, in a dog model, that the formation of thrombosis was associated with high shear stress, and the likelihood of thrombosis increased over time. Compared to catheters without a helical flow guide, those with a helical flow guide with a pitch of 2 mm demonstrate an increase in the average shear stress in the catheter by 48.5%, which may activate platelets under high shear stress, thereby increasing the risk of thrombosis. Therefore, we partially conclude that helical flow inducers are beneficial for reducing the risk of thrombosis in catheters based solely on the enhancement of helical flow. It is also essential to consider that the shear stress levels should not increase excessively while inducing helical flow. The calculation results show that when a helical flow inducer with a pitch of 8 mm (model-C) is placed inside the catheter lumen, the change in the average shear stress is relatively small; the high level of blood RTs (exceeding 0.15 s) and blood stagnation zones (velocity<0.1 m/s) were reduced by 15% and 27%, respectively. In the case of the same pitch, the increase in the diameter of the helical flow inducer (model-D to model-E) does not only contribute to reducing the blood retention area but will also increase the level of shear stress in the lumen. Therefore, the hemodynamic performance of the new design may be better than that of the traditional design; in particular, a Niagara^@^ catheter with a 8 mm pitch and 0.2 mm diameter inducer might be able to prevent the development of thrombosis.

While this study provides valuable insights into the design of helical flow inducers for dialysis catheters, several limitations should be noted. The simulations were conducted under idealized conditions, assuming laminar flow and neglecting patient-specific variations in blood properties. Future work should include more complex flow conditions, such as pulsatile flow and patient-specific blood rheology, to better predict the *in vivo* performance of these designs. Additionally, this study focused primarily on hemodynamic parameters within the catheter lumen, without considering the interactions between the catheter tip and the vascular wall, which could also significantly influence thrombosis risk. Future studies should explore these interactions to develop a more comprehensive understanding of how catheter design affects the overall performance. Furthermore, while the simulation results are promising, experimental validation is critical to confirm their applicability in clinical settings and to assess the generalizability of these findings across different patient populations. This remains an important limitation of the current study, and future work should prioritize experimental validation to validate the computational predictions.

## 5 Conclusion

In conclusion, the application of helical flow inducers to dialysis catheters presents a promising strategy for enhancing the hemodynamic performance and reducing thrombosis risk. Our findings suggest that larger pitch and larger diameter inducers are particularly effective in promoting helical flow, reducing residence time, and modulating shear stress, which could stabilize the blood flow, reduce the occurrence of flow disturbances, reduce embolisms, and promote the lysis of trapped clots. However, careful consideration must be given to the design of these inducers to balance the benefits of reduced thrombosis risk with the potential for shear-induced blood damage. Further research is warranted to validate these findings in clinical settings and to optimize catheter designs for broader clinical use.

## Data Availability

The raw data supporting the conclusions of this article will be made available by the authors, without undue reservation.

## References

[B1] Caro C. G. Cheshire N. J. Watkins N. (2005). Preliminary comparative study of small amplitude helical and conventional ePTFE arteriovenous shunts in pigs. J. R. Soc. Interface. 2, 261–266. 10.1098/rsif.2005.0044 16849184 PMC1629072

[B2] Carty G. Chatpun S. Espino D. M. (2016). Modeling blood flow through intracranial aneurysms: a comparison of Newtonian and non-Newtonian viscosity. J. Med. Biol. Eng. 36, 396–409. 10.1007/s40846-016-0142-z

[B3] Chen Y. Deng X. Shan X. Xing Y. (2018). Study of helical flow inducers with different thread pitches and diameters in vena cava. PLoS One 13, e0190609. 10.1371/journal.pone.0190609 29298357 PMC5752007

[B4] Cho S. Song R. Park S. C. Park H. S. Abbasi M. S. Lee J. (2021). Development of new hemodialysis catheter using numerical analysis and experiments. ASAIO J. 67, 817–824. 10.1097/mat.0000000000001315 33181539

[B5] Clark T. W. I. Isu G. Gallo D. Verdonck P. Morbiducci U. (2015). Comparison of symmetric hemodialysis catheters using computational fluid dynamics. J. Vasc. Interventional Radiology 26 (2), 252–259. 10.1016/j.jvir.2014.11.004 25645414

[B6] Fealy N. Kim I. Baldwin I. Schneider A. Bellomo R. (2018). A comparison of the Niagara™ and Medcomp™ catheters for continuous renal replacement therapy. Ren. Fail. 35, 308–313. 10.3109/0886022x.2012.757823 23356529

[B7] Gallo D. Steinman D. A. Bijari P. B. Morbiducci U. (2012). Helical flow in carotid bifurcation as surrogate marker of exposure to disturbed shear. J. Biomech. 45, 2398–2404. 10.1016/j.jbiomech.2012.07.007 22854207

[B8] Houston J. G. Gandy S. J. Milne W. Dick J. B. Belch J. J. Stonebridge P. A. (2004). Spiral laminar flow in the abdominal aorta: a predictor of renal impairment deterioration in patients with renal artery stenosis? Nephrol. Dial. Transpl. 19, 1786–1791. 10.1093/ndt/gfh238 15161949

[B9] Kwak D. Im Y. Nam H. Nam U. Kim S. Kim W. (2024). Analyzing the effects of helical flow in blood vessels using acoustofluidic-based dynamic flow generator. Acta Biomater. 117, 216–227. 10.1016/j.actbio.2024.01.021 38253303

[B10] Langston C. E. Eatroff A. E. (2018). Hemodialysis catheter-associated fibrin sheath in a dog. J. Vet. Emerg. Crit. Care San Ant. 28, 366–371. 10.1111/vec.12721 29763987

[B11] Lee S. W. Steinman D. A. (2007). On the relative importance of rheology for image-based CFD models of the carotid bifurcation. J. Biomech. Eng. 129, 273–278. 10.1115/1.2540836 17408332

[B12] Lihua W. Lan J. Aili J. (2022). Pathology of catheter-related complications: what we need to know and what should be discovered. J. Int. Med. Res. 50, 3000605221127890. 10.1177/03000605221127890 36268763 PMC9597033

[B13] Liu X. Sun A. Fan Y. Deng X. (2015). Physiological significance of helical flow in the arterial system and its potential clinical applications. Ann. Biomed. Eng. 43, 3–15. 10.1007/s10439-014-1097-2 25169424

[B14] Mareels G. Kaminsky R. Eloot S. Verdonck P. R. (2007). Particle image velocimetry–validated, computational fluid dynamics–based design to reduce shear stress and residence time in central venous hemodialysis catheters. Asaio J. 53, 438–446. 10.1097/mat.0b013e3180683b7c 17667228

[B15] Meirson T. Orion E. Avrahami I. (2015). Numerical analysis of venous external scaffolding Technology for saphenous vein grafts. J. Biomech. 48, 2090–2095. 10.1016/j.jbiomech.2015.03.011 25869720

[B16] Morbiducci U. Ponzini R. Rizzo G. Cadioli M. Esposito A. De Cobelli F. (2009). *In vivo* quantification of helical blood flow in human aorta by time-resolved three-dimensional cine phase contrast magnetic resonance imaging. Ann. Biomed. Eng. 37, 516–531. 10.1007/s10439-008-9609-6 19142728

[B17] Morbiducci U. Ponzini R. Rizzo G. Gadioli M. Esposito A. Moontevecchi F. M. (2011). Mechanistic insight into the physiological relevance of helical blood flow in the human aorta: an *in vivo* study. Biomech.Modeling Mechanobiol. 10, 339–355. 10.1007/s10237-010-0238-2 20652615

[B18] Owen D. G. de Oliveira D. C. Qian S. Green N. C. Shepherd D. E. T. Espino D. M. (2020). Impact of side-hole geometry on the performance of hemodialysis catheter tips: a computational fluid dynamics assessment. PLoS One 15 (8), e0236946. 10.1371/journal.pone.0236946 32764790 PMC7413473

[B19] Peng L. Qiu Y. Huang Z. Xia C. Dai C. Zheng T. (2017). Numerical simulation of hemodynamic changes in central veins after tunneled cuffed central venous catheter placement in patients under hemodialysis. Sci. Rep. 7, 15955. 10.1038/s41598-017-12456-7 29162830 PMC5698485

[B20] Pinelli F. Balsorano P. Mura B. Pittiruti M. (2021). Reconsidering the GAVeCeLT Consensus on catheter-related thrombosis, 13 years later. J. Vasc. Access 22, 501–508. 10.1177/1129729820947594 32772785

[B21] Stonebridge P. A. V ermassen F. Dick J. Belch J. J. Houston G. (2012). Spiral laminar flow prosthetic bypass graft: medium-term results from a first-in-man structured registry study. Ann. V. asc Surg. 26, 1093–1099. 10.1016/j.avsg.2012.02.001 22682930

[B22] Thapa S. Terry P. B. Kamdar B. B. (2016). Hemodialysis catheter-associated superior vena cava syndrome and pulmonary embolism: a case report and review of the literature. BMC Res. Notes 9, 233. 10.1186/s13104-016-2043-1 27107813 PMC4842288

[B23] Wong K. C. Büsen M. Benzinger C. Gäng R. Bezema M. Greatrex N. (2014). Effect of inflow cannula tip design on potential parameters of blood compatibility and thrombosis. Artif. Organs 38 (9), 810–817. 10.1111/aor.12369 25234762

[B24] Xu X. D. Han X. Yang Y. Li X. (2020). Comparative study on the efficacy of peritoneal dialysis and hemodialysis in patients with end-stage diabetic nephropathy. Pak J. Med. Sci. 36, 1484–1489. 10.12669/pjms.36.7.2901 33235561 PMC7674865

[B25] Yang C. Yang Z. Wang J. Wang H. Y. Su Z. Chen R. (2021). Estimation of prevalence of kidney disease treated with dialysis in China: a study of insurance claims data. Am. J. Kidney Dis. 77, 889–897.e1. 10.1053/j.ajkd.2020.11.021 33421457

[B26] Yang Y. Li Y. Liu C. Zhou J. Li T. Xiong Y. (2022). Hemodynamic analysis of the geometric features of side holes based on GDK catheter. J. Funct. Biomater. 13, 236. 10.3390/jfb13040236 36412877 PMC9680405

[B27] Zhan F. Fan Y. Deng X. (2010). Swirling flow created in a glass tube suppressed platelet adhesion to the surface of the tube: its implication in the design of small-caliber arterial grafts. Thromb. Res. 125, 413–418. 10.1016/j.thromres.2009.02.011 19304314

[B28] Zhang H. Lu H. Li W. Jiang G. Zou H. (2021). Expert consensus on the establishment and maintenance of native arteriovenous fistula. Chronic Dis. Transl. Med. 7, 235–253. 10.1016/j.cdtm.2021.05.002 34786543 PMC8579016

[B29] Zheng T. Wen J. Jiang W. Deng X. Fan Y. (2014). Numerical investigation of oxygen mass transfer in a helical-type artery bypass graft. Methods Biomech. Biomed. Eng. 17 (5), 549–559. 10.1080/10255842.2012.702764 22794110

